# Cepharanthine hydrochloride inhibits prostate cancer progression by modulating gut microbiota and metabolites

**DOI:** 10.3389/fphar.2025.1627656

**Published:** 2025-08-13

**Authors:** Hui Li, Xing Luo, Peng He, Zongming Dong, Yongming Jia, Bishao Sun, Ji Zheng, Jingzhen Zhu

**Affiliations:** ^1^ Department of Ultrasound, Second Affiliated Hospital, Army Medical University, Chongqing, China; ^2^ Department of Urology, Urologic Surgery Center, Xinqiao Hospital, Third Military Medical University (Army Medical University), Chongqing, China; ^3^ Department of Ultrasound Medicine & Ultrasonic Medical Engineering Key Laboratory of Nanchong City, Affiliated Hospital of North Sichuan Medical College, Nanchong, China

**Keywords:** prostate cancer, cepharanthine hydrochloride, gut microbiota, metabolites of gut microbiota, antibiotic cocktail

## Abstract

**Background:**

Cepharanthine Hydrochloride (CH) is widely used in clinical settings to alleviate leukopenia caused by various tumors following radiotherapy and chemotherapy. However, it remains unclear whether CH have an inhibitory effect on the progression of prostate cancer, and whether this effect is mediated by gut microbiota. To address this question, the present study constructed normal mouse models of prostate cancer, as well as antibiotic-treated mouse models of prostate cancer.

**Methods:**

CH were then administered via gavage to both groups of model mice. After treatment, the tumor sizes of the mice were measured, and feces, blood, and tumor tissues from both groups were collected for 16S rDNA, metabolomics, and transcriptomics sequencing analysis.

**Results:**

Results showed CH treatment significantly suppressed prostate cancer growth in mice without antibiotic cocktail pretreatment, but not in antibiotic-pretreated mice. 16S rRNA sequencing revealed distinct gut microbiota alterations in CH-Ctrl versus Ctrl/CH-ABX groups, with increased g_Blautia, g_*Lactobacillus*, g_Butyricicoccus and decreased g_Akkermansia abundances. Metabolomic analysis identified 240 and 123 differentially abundant metabolites in CH-Ctrl vs Ctrl and CH-ABX, respectively. RNA-seq detected 579 and 530 differentially expressed genes in CH-Ctrl vs Ctrl and CH-ABX, respectively. Correlation analysis of differential gut microbiota, metabolites, and genes suggested that CH might inhibit prostate cancer growth by increasing the relative abundance of g_Blautia, g_*Lactobacillus*, and g_Butyricicoccus, suppressing g_Akkermansia proliferation, enhancing Acetylglycine metabolite production, upregulating Ttpa, Gm14964, Shc3, Elovl4 gene expression, and downregulating Gm10531, Bc021767 gene expression.

**Conclusion:**

This study is the first to explore the potential mechanisms of gut microbiota-mediated CH treatment for prostate cancer, providing a scientific basis for the application of CH in PCa therapy.

## Background

Prostate cancer (PCa) is the second most prevalent malignancy in men globally, with an estimated 1.41 million new cases reported in 2022, representing 14% of all male cancers (Bray et al., 2024). This rising incidence is attributed to population aging and improved screening practices, yet therapeutic outcomes remain suboptimal, particularly in advanced stages (Siegel et al., 2025). In China, although the incidence of PCa remains lower than in Western countries, it is increasing rapidly. In 2022, China recorded 134,200 new PCa cases and 47,500 deaths (Han et al., 2024). Notably, a significant proportion of Chinese PCa patients are diagnosed at advanced stages, resulting in a lower 5-year survival rate compared to Western nations (Han et al., 2024). Current clinical treatments for PCa primarily rely on conventional approaches such as surgical resection, radiation therapy, endocrine therapy, and chemotherapy. In recent years, poly (ADP-ribose) polymerase (PARP) inhibitors, prostate-specific membrane antigen (PSMA)-targeted therapies, and immunotherapies have been gradually incorporated into clinical practice, yet their therapeutic efficacy remains suboptimal (Sekhoacha et al., 2022; Cornford et al., 2024; Tilki et al., 2024). Additionally, most PCa patients undergoing androgen deprivation therapy (ADT) eventually progress to castration-resistant prostate cancer (CRPC) despite achieving castrate serum testosterone levels, leading to poor treatment outcomes and unfavorable prognoses (Cai et al., 2023; Zhang et al., 2024). Furthermore, targeted delivery approaches utilizing small-molecule ligands and oligonucleotide-based therapeutics are being increasingly explored for PCa management (Li et al., 2024; Abdelaal et al., 2024). These challenges underscore the urgent need for innovative drug development to overcome existing therapeutic limitations and provide more effective, precision-based clinical solutions for PCa patients.

In recent years, with the deepening understanding of the tumor microenvironment, the gut microbiota, a vast and complex microbial community within the human body has gained increasing attention for its role in cancer development (Wong-Rolle et al., 2021). The gut microbiota not only helps maintain host immune function but also interacts with the host through the production of various metabolites, thereby influencing tumor initiation, progression, and metastasis (Park et al., 2022; Wong-Rolle et al., 2021; El and Garrett, 2023; Zitvogel et al., 2024). In prostate cancer (PCa), the impact of gut microbiota is particularly significant. Studies have shown that gut dysbiosis is closely associated with an increased risk of PCa, disease progression, and poor treatment response (Lachance et al., 2024; Fujita et al., 2022). For instance, alterations in the abundance of certain microbial species may promote PCa cell proliferation and invasion, while others may suppress PCa development by producing anti-inflammatory factors or antitumor metabolites (Pernigoni et al., 2023; Kustrimovic et al., 2023; Matsushita et al., 2021). Furthermore, the concept of the “gut-prostate axis” has been proposed, highlighting the intricate interactions between the gut and prostate and providing new insights into the pathogenesis of PCa (Lachance et al., 2024).

Natural products serve as a crucial source of novel drug candidates, and many have already been approved for anticancer therapy. Cepharanthine is a bisbenzylisoquinoline alkaloid extracted from plants of the Stephania genus (family Menispermaceae) and is commonly used in its hydrochloride salt form. It exhibits diverse pharmacological properties and clinical applications, including antiviral effects, leukocyte elevation, anti-inflammatory activity, and immunomodulation (Liu et al., 2023; Lu C. et al., 2023). Beyond these functions, cepharanthine has demonstrated antitumor activity across various cancer types (Wang et al., 2023; Lu Y. Y. et al., 2023; Bai et al., 2024). Cepharanthine hydrochloride (CH), synthesized by reacting cepharanthine with hydrochloric acid, retains the pharmacological activity of cepharanthine while potentially offering improved solubility and stability due to its hydrochloride form, making it more suitable for clinical formulations (Guan et al., 2025). Given the significant therapeutic potential of CH as an anticancer agent, elucidating its mechanisms of action and therapeutic effects represents a critical avenue for future cancer research.

To investigate how CH inhibits PCa growth, we established a mouse model and analyzed tumor development under CH treatment. Using 16S rDNA amplicon sequencing, untargeted metabolomics, and transcriptome sequencing technologies, we analyzed the gut microbiota composition, intestinal metabolites, and tumor transcriptome profiles in mice. Our findings reveal CH’s regulatory effects on gut microbiota diversity and composition, while elucidating the potential association between microbiota alterations and PCa growth inhibition. This study aims to provide scientific evidence for CH’s therapeutic application in PCa treatment, while offering novel insights into understanding the role of the “gut-prostate axis” in PCa pathogenesis.

## Materials and methods

### Cell lines and cell cultures

The murine PCa cell line RM-1 was obtained from Shanghai Zhong Qiao Xin Zhou Biotechnology Co., Ltd. and authenticated by STR profiling. RM-1 cells were cultured in RPMI-1640 medium (Gibco, United States) supplemented with 10% fetal bovine serum (FBS) (Gibco, United States), 1% penicillin, and 1% streptomycin (Gibco, United States). The cell line was maintained in a humidified incubator at 37°C with 5% CO_2_.

### Establish a subcutaneous tumor model of PCa in mice

All C57 mice were obtained from Beijing Vital River Laboratory Animal Technology Co., Ltd. All animal procedures and experimental protocols were conducted in accordance with the Guide for the Care and Use of Laboratory Animals and approved by the Laboratory Animal Welfare and Ethics Committee of Army Medical University, China (Approval No.: AMUWEC2020186). Every effort was made to minimize animal suffering and reduce the number of animals used. Male C57 mice (6 weeks old) were used in the experiments. The mice were randomly assigned to the Ctrl, CH-ABX, and CH-Ctrl groups (n = 10 per group) and housed individually under standard 12-h light/dark conditions. Mice in the CH-ABX group received antibiotic treatment for 2 weeks prior to the experiment via drinking water containing 0.2 g/L ampicillin, neomycin, metronidazole, and 0.1 g/L vancomycin daily. Approximately 2 × 10^6^ RM-1 cells were subcutaneously injected into the inner thigh of each mouse. On day 7 after establishing the tumor-bearing mouse model, CH (Chengdu MUST Bio-technology Co., Ltd, China) was dissolved in water and administered daily by gavage at 0.2 mg CH per mouse in the CH-ABX and CH-Ctrl groups, while the Ctrl group received water alone. After 4 weeks, the mice were weighed and euthanized via sodium pentobarbital injection. Subcutaneous tumors and intestinal content samples were collected, and tumor weight was measured.

### 16S rRNA sequencing and analysis

DNA was extracted from samples in the Ctrl, CH-ABX, and CH-Ctrl groups using a fecal DNA extraction kit (DP712, Tiangen Company, Beijing, China), with 10 replicates for each. Quality assessment of the DNA was done on 1% agarose gels. The V3-V4 region of the 16S-rDNA gene was targeted for amplification via PCR with specific primers: 341F: CCTACGGGNGGCWGCAG; 806R: GGACTACHVGGGTATCTAAT, each with a unique eight-base barcode sequence. PCR was conducted in 30 μL volumes containing 15 μL of Phusion^®^ High-Fidelity PCR Master Mix, 0.2 μM of primers, and 10 ng of DNA template. The thermal profile included an initial denaturation at 98°C for 1 min, followed by 30 cycles of 98°C for 10 s, 50°C for 30 s, and 72°C for 30 s, concluding with a final extension at 72°C for 5 min. PCR products were visualized on 2% agarose gels and purified using the GeneJET Gel Extraction Kit. Sequencing libraries were prepared with the Illumina TruSeq DNA PCR-Free Library Preparation Kit and indexed. Sequencing was performed on the Illumina Hiseq platform by Genecloud Co. Ltd, Chongqing, China. QIIME software package 2 (version 2020.2) was used for sequence analysis. Operational taxonomic units (OTUs) were determined at a similarity threshold of ≥97% using the MOTHUR pipeline, with the most abundant tag sequence chosen as representative. Alpha-diversity metrics such as Observed OTU, Shannon, and Simpson indices were calculated with the “vegan” R package. Beta-diversity was analyzed using Principal coordinates analysis (PCoA) and Partial Least Squares Discriminant Analysis (PLS-DA) through R. Taxonomic comparisons at phylum and genus levels were made using the Wilcoxon rank-sum test. LEfSe method was employed for differential abundance analysis of bacterial communities, with Kruskal–Wallis rank-sum test for significance (padj <0.05) and LDA score ≥2.5 indicating substantial effect size. Metabolic pathways were analyzed using the Kyoto Encyclopedia of Genes and Genomes (KEGG) database, considering pathways enriched when padj <0.05. To enhance statistical robustness and reduce the probability of Type I errors, a false discovery rate (FDR) correction was applied. Correlation between microbial flora and metabolites was assessed with Pearson’s correlation coefficient via R’s mixOmics package.

### Non-targeted metabolomics analysis

Prior to metabolite extraction, samples were weighed and lyophilized. They were then ground in a 2 mL Eppendorf tube with a 5 mm tungsten bead for 60 s at 65 Hz using a Grinding Mill. The extraction process involved a 1 mL mixture of methanol, acetonitrile, and water (in a ratio of 2:2:1), followed by an hour of ultrasonication in an ice bath. Afterward, the samples were chilled at −20°C for an hour and centrifuged at 14,000 g for 20 min at 4°C. The supernatants were collected and evaporated to dryness under vacuum conditions. Metabolomics profiling was conducted using a UPLC-ESI-Q-Orbitrap-MS system, which included a UHPLC Shimadzu Nexera X2 LC-30AD from Shimadzu, Japan, and a Q-Exactive Plus from Thermo Scientific, San Jose, United States. For LC separation, an ACQUITY UPLC^®^ HSS T3 column (2.1 × 100 mm, 1.8 μm) from Waters, Milford, MA, United States was utilized. The flow rate was set at 0.3 mL/min with a mobile phase consisting of A: 0.1% FA in water and B: 100% acetonitrile (ACN). The gradient elution started at 0% buffer B for 2 min, increased linearly to 48% over 4 min, then to 100% in another 4 min, maintained this level for 2 min, and finally decreased back to 0% buffer B in 0.1 min with a 3-min reequilibration period. ESI in both positive and negative modes was used for MS data collection. The HESI source parameters were as follows: Spray Voltage of 3.8 kv (positive) and 3.2 kv (negative); Capillary Temperature of 320°C; Sheath Gas (nitrogen) flow at 30 arb; Aux Gas flow at five arb; Probe Heater Temp at 350°C; S-Lens RF Level at 50. The mass range for full MS scans was set from 70 to 1050 Da with a resolution of 70,000 at m/z 200 and 17,500 for MS/MS scans. The injection time was set to 100 ms for MS and 50 ms for MS/MS. The isolation window for MS2 was set to 2 m/z with collision energies of 20, 30, and 40 for fragmentation. Raw MS data were processed using MS-DIAL for peak alignment and area extraction. R (version: 4.0.3) and its packages were employed for multivariate data analysis and modeling with Pareto scaling for mean-centering. Principal component analysis (PCA) was used to construct models, and VIP scores were calculated to assess variable significance with values over 1 considered significant. Metabolites with VIP values above 1.0 and p values below 0.05 were deemed statistically significant. Fold change was determined by calculating the logarithm of the average mass response ratio between two classes. Differential metabolites were subjected to cluster analysis using R packages. Perturbed biological pathways were identified through KEGG pathway analysis with differential metabolite data using the KEGG database and Fisher’s exact test with FDR correction for multiple testing. To enhance statistical robustness and reduce the probability of Type I errors, a FDR correction was applied. Pathways with padj <0.05 were considered statistically significant after enrichment analysis.

### Transcriptome sequencing analysis

Total RNA was extracted utilizing the TRIzol reagent (Invitrogen, CA, United States) following the manufacturer’s guidelines. Subsequently, libraries were prepared with the VAHTS Universal V6 RNA-seq Library Prep Kit as directed by the manufacturer. Transcriptome sequencing and analysis were executed by OBiO Technology Corp., Ltd. (Shanghai, China). Sequencing was performed on the Illumina Novaseq 6000 platform, yielding 150 bp paired-end reads. Initial processing of raw reads was conducted using fastp (v0.22.0) to eliminate low-quality sequences and retain clean reads. These clean reads were then aligned to the reference genome with HISAT2 (v2.1.0). Gene expression levels were quantified by calculating FPKM values, and read counts for each gene were obtained through feature Counts. PCA was performed on samples using standardized expression values to reduce data complexity, thoroughly explore relationships among samples and variation magnitude, and assess sample reproducibility. To enhance statistical robustness and reduce the probability of Type I errors, a FDR correction was applied. Differential expression analysis was conducted using DESeq2, with significance defined as padj <0.05. To illustrate gene expression patterns across different groups and samples, hierarchical cluster analysis of differentially expressed genes (DEGs) was performed using R (v3.2.0). Additionally, GO and KEGG pathway enrichment analyses of DEGs were conducted based on the hypergeometric distribution to identify significantly enriched terms, utilizing R (v3.2.0) for each analysis.

### Multi-omics joint analysis

Spearman correlation coefficients were used to perform pairwise correlation analyses among differential bacterial genera, genes, and metabolites, and a correlation network was constructed to elucidate the relationships between differential bacterial genera, metabolites, and genes. We applied FDR correction to minimize the likelihood of Type I errors to the greatest extent possible.

### Statistical analysis

The data are presented as the mean ± standard deviation (SD). We employed SPSS software (SPSS 26.0, IBM Corporation, United States) for statistical analyses and GraphPad Prism (version 8.0; GraphPad Software, LLC, United States) to create graphs. To compare the data before and after treatment, paired t - tests were carried out. For comparisons among multiple groups, one - way ANOVA was utilized. When it came to further comparing two groups, the least significant difference method was applied. For non - normally distributed data, the Mann - Whitney U test was used to evaluate group differences. Statistical significance was defined as a P - value of less than 0.05.

## Results

### CH treatment can significantly inhibit the growth of mouse PCa

**FIGURE 1:**
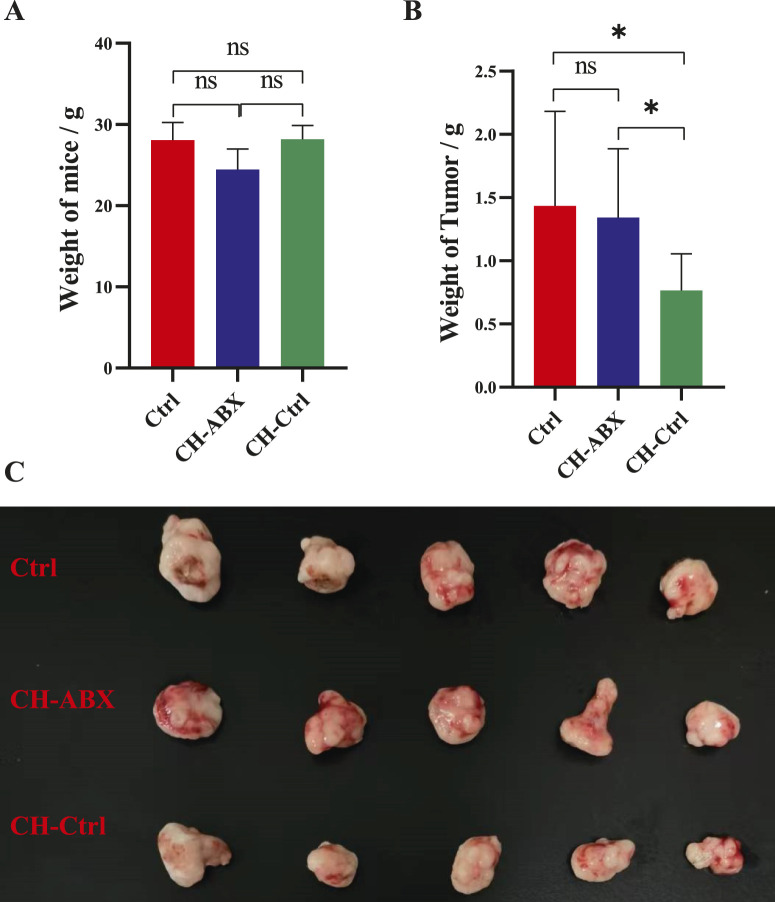
CH treatment significantly inhibited the growth of prostate tumors in mice. **(A)** Comparison of body weight among the three groups after treatment; **(B)** Comparison of tumor weight among the three groups after treatment; **(C)** Gross morphology of prostate tumors from the three groups after treatment. ^ns^ p > 0.05 *p ≤ 0.05, **p < 0.01, ***p < 0.001, n = 5.

The body weight and tumor weight of mice in the Ctrl, CH-Ctrl, and CH-ABX groups were measured and grossly examined. We found no significant differences in body weight among the groups (Figure 1A). However, the tumor weight in the CH-Ctrl group was significantly lower compared to both the Ctrl and CH-ABX groups. Although the CH-ABX group showed a decreasing trend in tumor weight relative to the Ctrl group, the difference was not statistically significant (Figures 1B,C). These results suggest that the gut microbiota may mediate the inhibitory effect of CH on PCa growth.

### The effect of CH treatment on the gut microbiota of PCa model mice

**FIGURE 2:**
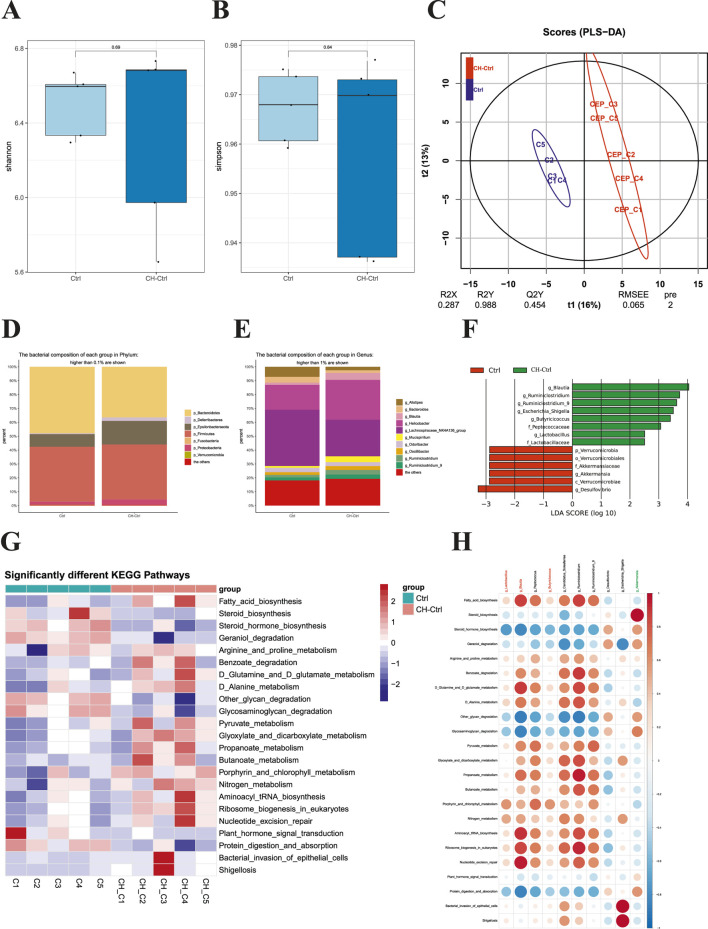
Comparison of gut microbiota between the Ctrl and CH-Ctrl groups. **(A,B)** Comparison of α-diversity indices between groups; **(C)** PLS-DA analysis of the two groups; **(D)** Relative abundance bar plot of gut microbiota at phylum level (>0.1% abundance); **(E)** Relative abundance bar plot of gut microbiota at genus level (>1% abundance); **(F)** LEfSe analysis showing significantly discriminant taxa between groups (LDA score ≥2.5); **(G)** Heatmap of significantly different KEGG pathways between groups; **(H)** Correlation heatmap between significantly different genus-level microbiota and metabolic pathways. ^ns^ p > 0.05 *p ≤ 0.05, **p < 0.01, ***p < 0.001, n = 5.

We next performed 16S rDNA amplicon sequencing analysis on the gut microbiota of each group. The results showed no significant differences in Shannon and Simpson α-diversity indices between the Ctrl and CH-Ctrl groups (Figures 2A,B). PLS-DA analysis revealed clear inter-group separation and intra-group clustering between the Ctrl and CH-Ctrl groups (Figure 2C). At the phylum level (relative abundance >0.1%) and genus level (relative abundance >1%), the overall structure of gut microbiota showed minimal changes between the two groups, though significant alterations were observed in the relative abundance of specific phyla and genera (Figures 2D,E). Specifically, compared with the Ctrl group, the CH-Ctrl group exhibited decreased relative abundance of p_Bacteroidetes but increased relative abundance of p_Deferribacteres, p_Epsilonbacteraeota, and p_Proteobacteria at the phylum level (Figure 2D). At the genus level, the CH-Ctrl group showed reduced relative abundance of g_Alistipes, g_*Bacteroides*, and g_Lachnospiraceae_NK4A136_group, while demonstrating increased relative abundance of g_Blautia, g_*Helicobacter*, g_Mucispirillum, g_Ruminiclostridium, and g_Ruminiclostridium_9 compared to the Ctrl group (Figure 2E). Linear discriminant analysis (LDA) with an effect size threshold (LDA score >2.5) identified significantly different microbial biomarkers between the two groups (Figure 2F). Subsequent KEGG pathway analysis of gut microbiota revealed 23 significantly different metabolic pathways between the groups (Figure 2G). Finally, correlation analysis between the significantly different gut microbiota and KEGG metabolic pathways in Ctrl and CH-Ctrl groups generated a positive/negative correlation map (Figure 2H).

**FIGURE 3:**
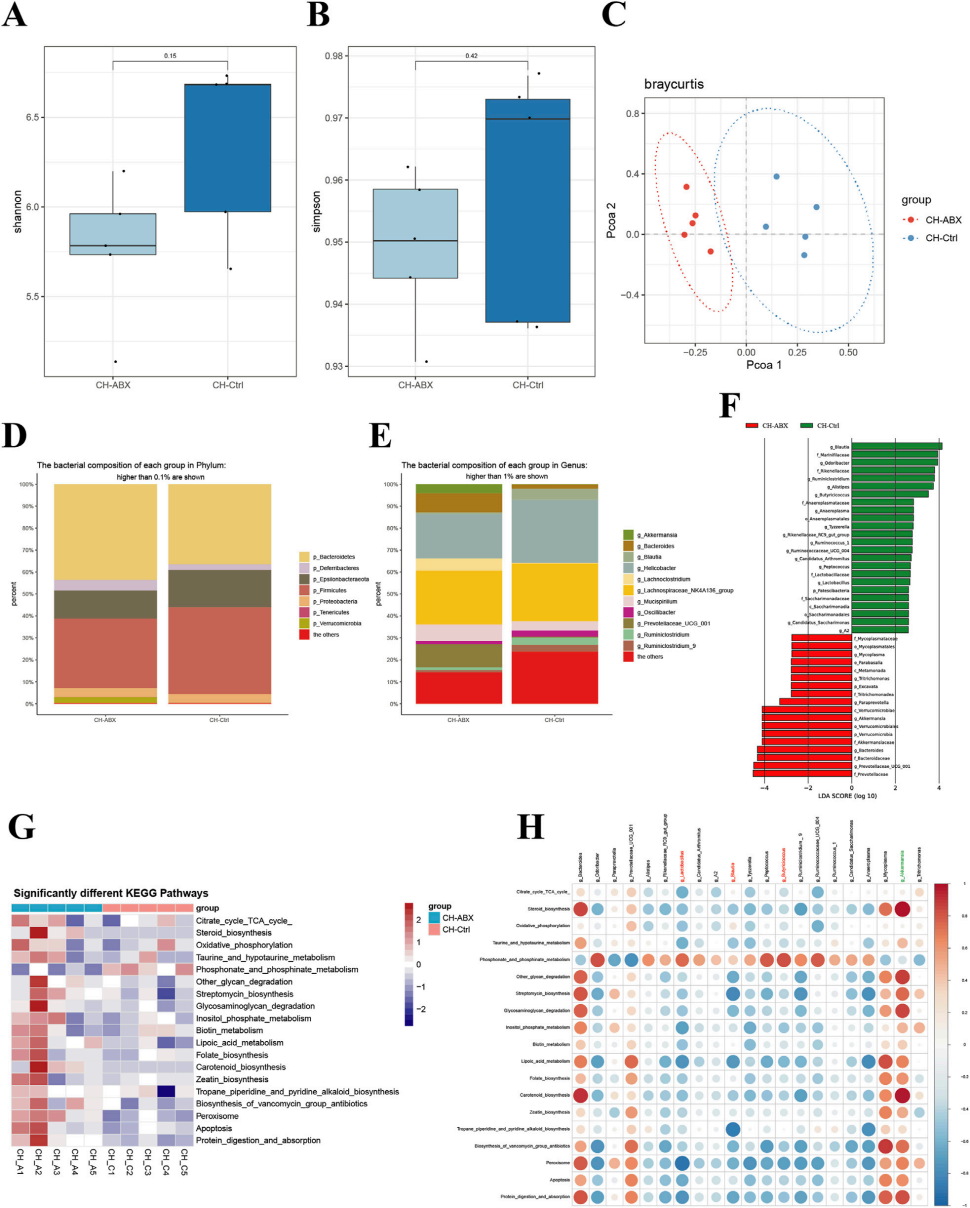
Comparison of gut microbiota between the Ctr-ABX and CH-Ctrl groups. **(A,B)** Comparison of α-diversity indices between groups; **(C)** Principal coordinates analysis (PCoA) of the two groups; **(D)** Bar plot showing relative abundance of gut microbiota at phylum level (>0.1% abundance); **(E)** Bar plot showing relative abundance of gut microbiota at genus level (>1% abundance); **(F)** LEfSe analysis of significantly discriminant taxa between groups (LDA score ≥2.5); **(G)** Heatmap of significantly different KEGG pathways between groups; **(H)** Heatmap showing correlations between significantly different genus-level microbiota and metabolic pathways. ^ns^ p > 0.05 *p ≤ 0.05, **p < 0.01, ***p < 0.001, n = 5.

Comparative analysis between CH-Ctrl and CH-ABX groups revealed increased Shannon and Simpson α-diversity indices in the CH-Ctrl group, though without statistical significance (Figures 3A,B). Principal Coordinates Analysis (PCoA) demonstrated distinct inter-group separation and intra-group clustering between CH-ABX and CH-Ctrl groups (Figure 3C). Significant structural alterations were observed at both phylum (relative abundance >0.1%) and genus (relative abundance >1%) levels, accompanied by marked changes in relative abundance of specific taxa (Figures 3D,E). At the phylum level, CH-Ctrl group exhibited reduced or undetectable levels of p_Bacteroidetes, p_Deferribacteres, and p_Verrucomicrobia, while showing increased relative abundance of p_Epsilonbacteraeota and p_Firmicutes compared to CH-ABX group (Figure 3D). Genus-level analysis demonstrated decreased or absent g_Akkermansia, g_*Bacteroides*, g_Lachnoclostridium, g_Mucispirillum, and g_Prevotellaceae_UGG_001, contrasted with elevated levels of g_Blautia, g_*Helicobacter*, g_Oscillibacter, g_Ruminiclostridium, and g_Ruminiclostridium_9 in CH-Ctrl group (Figure 3E). Linear discriminant analysis (LDA) with an effect size threshold (LDA score >2.5) identified significantly differential microbial biomarkers between the groups (Figure 3F). KEGG pathway analysis revealed 19 significantly different metabolic pathways (Figure 3G). Correlation analysis between these differential gut microbiota and metabolic pathways generated a comprehensive positive/negative correlation network (Figure 3H).

Comprehensive analysis integrating the relative abundance trends of differential microbiota between CH-Ctrl vs Ctrl and CH-Ctrl vs CH-ABX groups suggests that CH may influence PCa progression by increasing the relative abundance of g_Blautia, g_*Lactobacillus*, and g_Butyricicoccus while decreasing g_Akkermansia abundance.

### The effect of CH treatment on intestinal metabolites in PCa model mice

**FIGURE 4:**
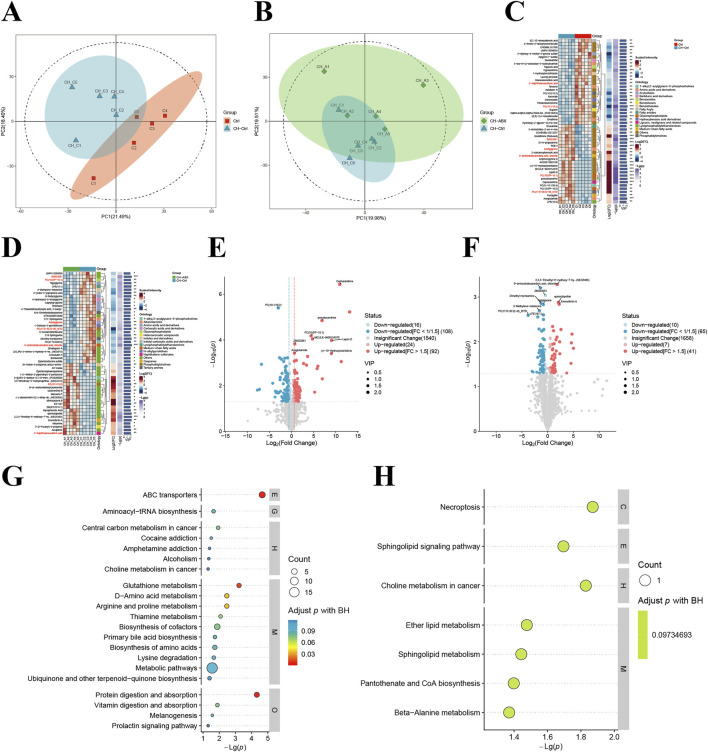
Comparison of intestinal metabolites between groups. **(A,B)** Principal component analysis (PCA) between groups; **(C,D)** Hierarchical clustering heatmap of differential metabolites between groups; **(E,F)** Volcano plots of inter group metabolite differences (differential metabolites screened by p-value + VIP criteria, with red indicating upregulation and blue indicating downregulation); **(G)** KEGG pathway bubble plot for CH-Ctrl vs. Ctrl differential metabolites (bubble size represents the number of differential metabolites annotated to each pathway, color intensity corresponds to adjusted p-values with blue-to-red gradient indicating increasing significance); **(H)** KEGG pathway bubble plot for CH-ABX vs. CH-Ctrl differential metabolites (with identical representation scheme as panel **(G)**). ^ns^ p > 0.05 *p ≤ 0.05, **p < 0.01, ***p < 0.001, n = 5.

Our analysis of untargeted metabolomics in intestinal contents from PCa mice across groups revealed that the CH-Ctrl group showed distinct separation from the Ctrl group along the PC1 axis (21.49%) (Figure 4A) and from the CH-ABX group along the PC2 axis (19.51%) (Figure 4B). Volcano plot analysis identified 240 and 123 significantly differential metabolites when comparing CH-Ctrl with Ctrl and CH-ABX groups, respectively (Figures 4E,F). Heatmap clustering of the top 50 differential metabolites demonstrated that, compared to the Ctrl group, the CH-Ctrl group exhibited 24 significantly upregulated and 26 downregulated metabolites (Figure 4C). Similarly, relative to the CH-ABX group, there were 22 upregulated and 28 downregulated metabolites in the CH-Ctrl group (Figure 4D). The metabolites consistently upregulated in CH-Ctrl compared to both Ctrl and CH-ABX groups were ZM323881, acetylglycine, PC(17:0-20:3)-d5_ISTD, 6-aminododecanedioic acid, and chloride. Conversely, PC (18:1/18:2) and 2-naphthalenesulfonic acid were consistently downregulated. KEGG pathway enrichment analysis of these differential metabolites identified choline metabolism in cancer as the common significantly enriched pathway between CH-Ctrl versus both Ctrl and CH-ABX comparisons (Figures 4G, H).

### The effect of CH treatment on the transcriptome expression of mouse PCa

**FIGURE 5:**
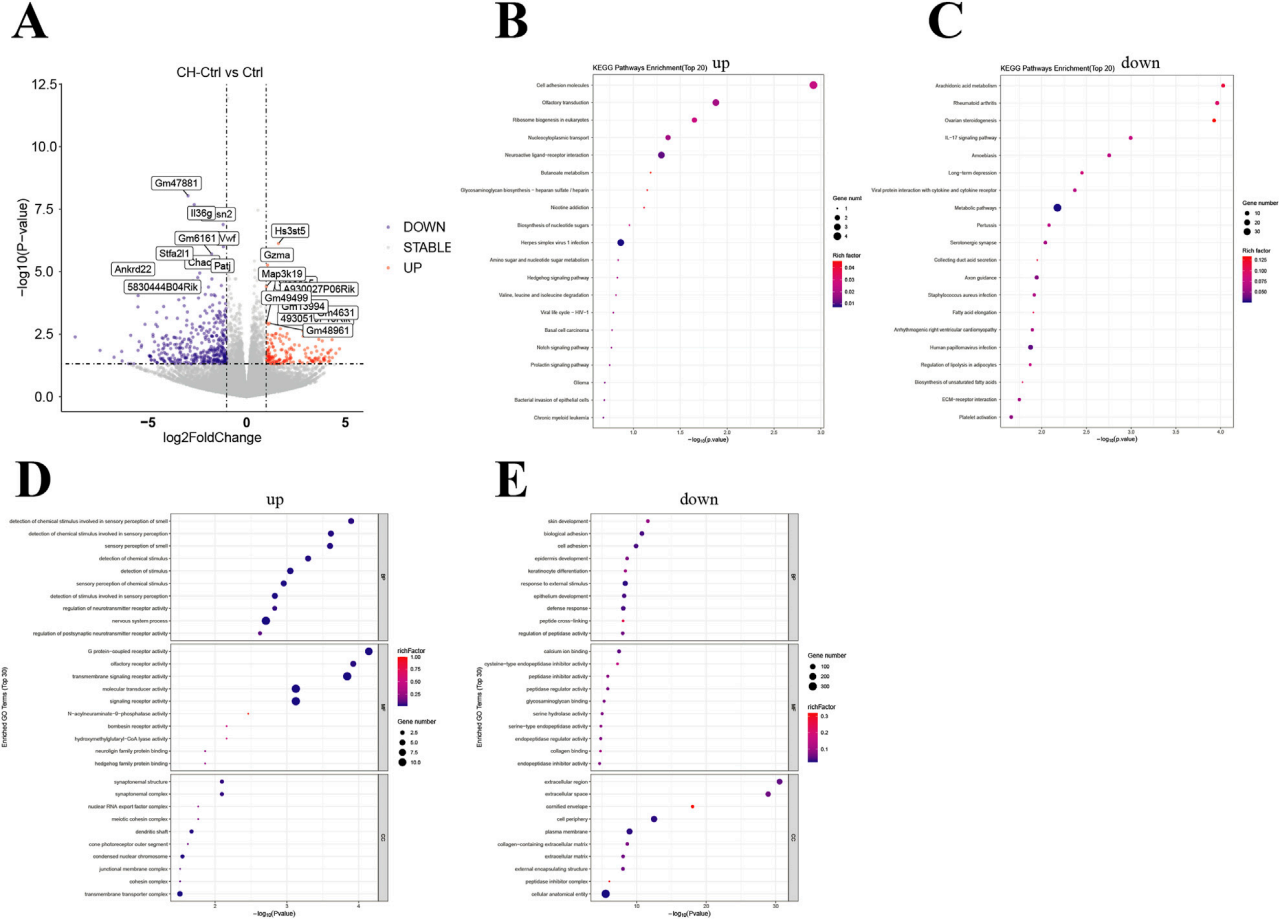
Analysis of differential genes between the CH-Ctrl vs. Ctrl groups **(A)** Volcano plot of differentially expressed genes, with the x-axis representing fold change in gene expression and the y-axis indicating the statistical significance of expression changes. Data points represent individual genes: gray denotes non-significant differences, red indicates significantly upregulated genes, and blue represents significantly downregulated genes; **(B)** Bubble plot of the top 20 significantly enriched KEGG pathways (upregulated); **(C)** Bubble plot of the top 20 significantly enriched KEGG pathways (downregulated); **(D)** Bubble plot of the top 30 significantly enriched GO terms (upregulated); **(E)** Bubble plot of the top 30 significantly enriched GO terms (downregulated). In all bubble plots, the Rich factor is represented by point color (gradient from low to high values corresponding to blue to red), while the size of each point reflects the number of differentially expressed genes annotated to each KEGG pathway/GO term. ^ns^ p > 0.05 *p ≤ 0.05, **p < 0.01, ***p < 0.001, n = 5.

**FIGURE 6:**
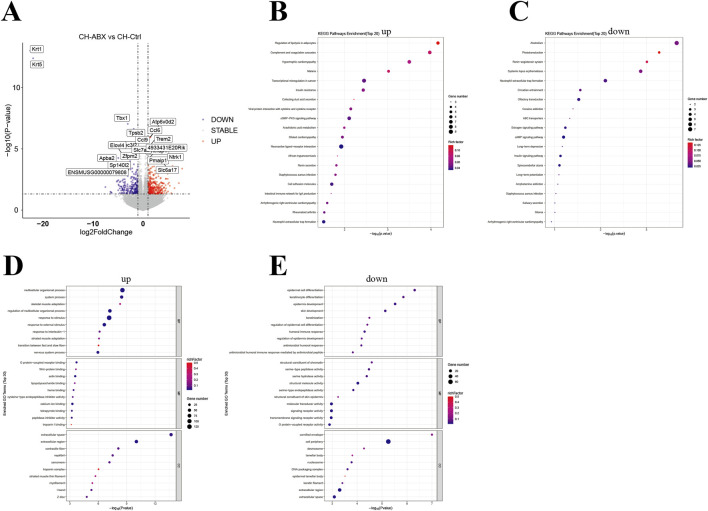
Analysis of differential genes between the CH-ABX vs. CH-Ctrl groups. **(A)** Volcano plot of differentially expressed genes, with the x-axis representing fold change in gene expression and the y-axis indicating the statistical significance of expression changes. Data points represent individual genes: gray denotes non-significant differences, red indicates significantly upregulated genes, and blue represents significantly downregulated genes; **(B)** Bubble plot of the top 20 significantly enriched KEGG pathways (upregulated); **(C)** Bubble plot of the top 20 significantly enriched KEGG pathways (downregulated); **(D)** Bubble plot of the top 30 significantly enriched GO terms (upregulated); **(E)** Bubble plot of the top 30 significantly enriched GO terms (downregulated). In all bubble plots, the Rich factor is represented by point color (gradient from low to high values corresponding to blue to red), while the size of each point reflects the number of differentially expressed genes annotated to each KEGG pathway/GO term. ^ns^ p > 0.05 *p ≤ 0.05, **p < 0.01, ***p < 0.001, n = 5.

Simultaneously, we performed transcriptome sequencing analysis on PCa tissues from the mice. Overall, the CH-Ctrl group exhibited 166 significantly upregulated and 413 downregulated genes compared to the Ctrl group (Figure 5A), while showing 312 upregulated and 218 downregulated genes relative to the CH-ABX group (Figure 6A). KEGG pathway enrichment analysis of the differentially expressed genes revealed that, compared to both Ctrl and CH-ABX groups, the CH-Ctrl group showed concurrent significant enrichment in the top 20 KEGG pathways for both increased pathways (Cell adhesion molecules and Neuroactive ligand-receptor interaction; Figures 5B, 6B) and decreased pathways (*Staphylococcus aureus* infection and Arrhythmogenic right ventricular cardiomyopathy; Figures 5C, 6C). Subsequent GO functional enrichment analysis demonstrated that, relative to both Ctrl and CH-ABX groups, the CH-Ctrl group displayed simultaneous significant enrichment in increased GO terms (nervous system process; Figures 5D, 6D) and decreased GO terms (epidermis development, cell periphery, extracellular region, and extracellular space; Figures 5E, 6E).

### CH modulates the gut microbiota, thereby altering intestinal metabolites which subsequently influence transcriptome expression

**FIGURE 7:**
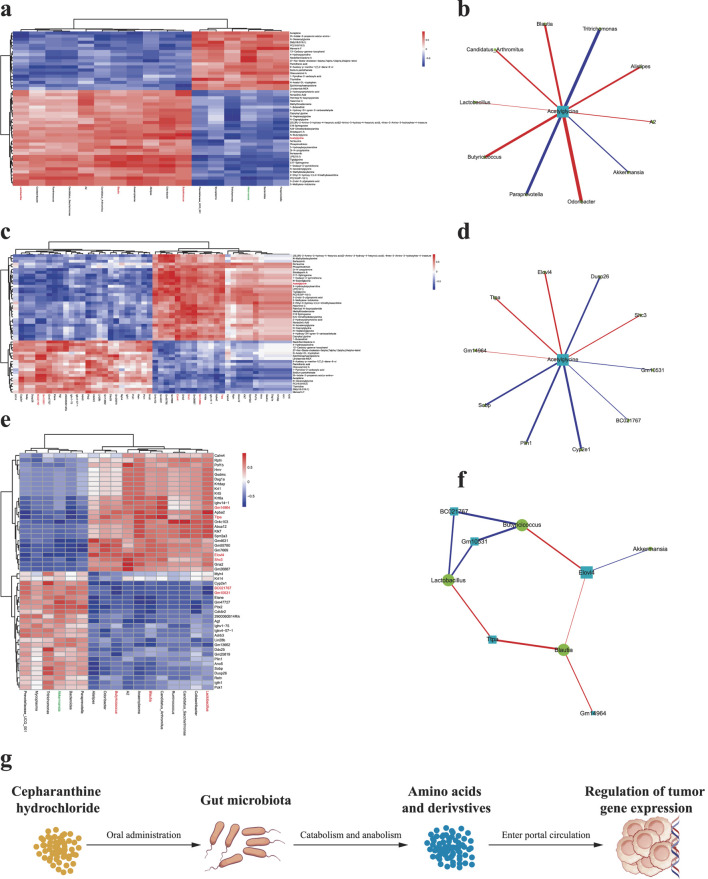
Pairwise correlation analysis among differential gut microbiota, intestinal metabolites, and genes **(a)** Correlation analysis between differential gut microbiota and intestinal metabolites; **(b)** Correlation network of differential gut microbiota and metabolites; **(c)** Correlation analysis between differential intestinal metabolites and genes; **(d)** Correlation network of differential metabolites and genes; **(e)** Correlation analysis between differential gut microbiota and genes; **(f)** Correlation network of differential microbiota and genes. Red markers indicate significantly correlated differential gut microbiota, metabolites, and genes potentially involved in CH-mediated suppression of PCa growth. **(g)** Schematic that the “microbiota-metabolome-transcriptome” axis. ^ns^ p > 0.05 *p ≤ 0.05, **p < 0.01, ***p < 0.001, n = 5.

Through comprehensive correlation analysis of significantly altered gut microbiota, intestinal metabolites, and transcriptome data - combined with our previous findings - we identified key differential gut microbes, metabolites, and genes potentially involved in CH-mediated suppression of PCa growth. CH treatment significantly increased the relative abundance of g_Blautia, g_*Lactobacillus*, and g_Butyricicoccus while inhibiting g_Akkermansia proliferation. This microbial shift enhanced Acetylglycine production, consequently upregulating Ttpa, Gm14964, Shc3, and Elovl4 gene expression while downregulating Gm10531 and Bc021767 (Figure 7).

## Discussion

In recent years, the “gut-tumor axis” has emerged as a research hotspot in oncology. Numerous studies have demonstrated the crucial role of gut microbiota in the initiation, progression, and treatment of various cancers (Zitvogel et al., 2024; Sepich-Poore et al., 2021; Fujita et al., 2023). As a leukocyte stimulator, CH has previously been explored for cancer treatment, including PCa (Wang et al., 2023; Guan et al., 2025). This study investigated CH’s inhibitory effects on PCa growth in mice and its underlying mechanisms. Our findings reveal that CH treatment significantly suppresses PCa growth in mice, and this antitumor effect may be mediated through its regulation of gut microbiota composition (Figure 7g).

Our study revealed that the CH-Ctrl group exhibited significantly reduced tumor weight compared to both Ctrl and CH-ABX groups, while no significant difference was observed between CH-ABX and Ctrl groups. The marked attenuation of CH’s antitumor efficacy following antibiotic-induced depletion of gut microbiota strongly suggests that the gut microbiota play a critical mediating role in CH-induced suppression of prostate cancer (PCa) growth. Antibiotic cocktail pretreatment disrupts intestinal microbiota homeostasis and metabolic functional activity in mice, thereby impairing host physiological and metabolic functions. While such effects may occur independently of microbial depletion, they primarily influence host health through microbiota-mediated mechanisms (Vliex et al., 2024; Tao et al., 2020). These findings align with the growing body of research highlighting the importance of gut microbiota in tumorigenesis, progression, and therapeutic response (Liang et al., 2025; Qu et al., 2023; Laborda-Illanes et al., 2020).

To investigate the changes in gut microbiota of PCa mice after CH treatment, we performed 16S rDNA amplicon sequencing analysis on the three experimental groups. The results showed that CH treatment significantly altered both the structure and function of gut microbiota. Notably, at the phylum level, our findings demonstrate that CH treatment significantly increased the abundance of Proteobacteria. While elevated Proteobacteria levels are recognized as a microbial signature of dysbiosis (Shin et al., 2015), this observation appears contradictory to the established consensus that dysbiosis generally promotes tumor progression (Xie and Liu, 2024). The underlying mechanisms remain unclear - whether CH or its secondary metabolites directly stimulate Proteobacteria proliferation, or alternatively, suppress competing bacterial populations leading to relative enrichment of Proteobacteria. These intriguing possibilities warrant further investigation in future studies. At the genus level, CH treatment markedly increased the relative abundances of g_Blautia, g_*Lactobacillus*, and g_Butyricicoccus while significantly decreasing g_Akkermansia abundance. The g_Blautia is a bacterial genus known to produce short-chain fatty acids (SCFAs, such as butyrate), which have demonstrated anti-inflammatory, immunomodulatory, and tumor-suppressive effects (Holmberg et al., 2024; Shoji et al., 2022), both g_*Lactobacillus* and g_Butyricicoccus are also closely associated with gut health and immune regulation (Chen et al., 2023; Chang et al., 2022; Chang et al., 2020). In contrast, g_Akkermansia, recognized for its mucin-degrading capacity, was found to be significantly increased in DSS-treated mice, leading to exacerbated inflammation (Li et al., 2018). Several studies have established a strong correlation between g_Akkermansia overgrowth and inflammatory responses in both colon and liver (Chen et al., 2025). Notably, emerging evidence suggests g_Akkermansia plays a critical role in tumor immunotherapy response (Zhu et al., 2024), highlighting the need for further research to elucidate its complex functions in tumor development. Furthermore, KEGG pathway analysis revealed that CH treatment induced significant alterations in microbial metabolic pathways. These differentially expressed metabolic pathways may be closely associated with both the gut microbiota modifications and the anti-tumor effects of CH.

Previous studies have demonstrated that CEP exhibits poor oral absorption and low absolute bioavailability in rats, mice, rabbits, dogs, and humans (Hao et al., 2010; Desgrouas et al., 2014; Deng et al., 2017; Li et al., 2022; Xia et al., 2023). Therefore, investigating the catabolism of CH by gut microbiota in the intestine is of great importance. Comparative analysis of intestinal metabolites revealed that the CH-Ctrl group exhibited significantly elevated levels of ZM323881, acetylglycine, PC(17:0-20:3)-d5_ISTD, 6-aminododecanedioic acid, and chloride, along with decreased levels of PC(18:1/18:2) and 2-naphthalenesulfonic acid relative to both Ctrl and CH-ABX groups. KEGG pathway enrichment analysis identified choline metabolism in cancer as the common pathway for these differential metabolites. Notably, aberrant choline metabolism represents a hallmark of cancer (Amstalden et al., 2010). Our findings suggest CH may inhibit PCa growth by modulating gut microbiota to influence choline metabolic pathways. Integrated analysis of differential gut microbiota and metabolites identified acetylglycine as a key microbial-derived metabolite potentially mediating CH’s effects. Existing evidence demonstrates that N-acetylglycine: shows the strongest inverse correlation with HCC risk (Sanchez et al., 2024). Exerts protective effects against obesity and related metabolic disorders (Su et al., 2023). Mediates microbiome-dependent weight gain post-smoking cessation (Fluhr et al., 2021). Reduces obesity-associated Trem2+ macrophages and modifies immunometabolic signaling in high-fat diet models (Sanchez et al., 2024). These findings suggest N-acetylglycine may confer protection against PCa through immunometabolic regulation of obesity-related pathways.

Transcriptome sequencing analysis revealed that the CH-Ctrl group exhibited significant alterations in gene expression compared to both Ctrl and CH-ABX groups. Through integrated analysis of differential gut microbiota, intestinal metabolites, and transcriptome profiles, we identified upregulated genes (Ttpa, Gm14964, Shc3, Elovl4) and downregulated genes (Gm10531, Bc021767). These gene expression changes likely represent indirect effects of CH on PCa via modulation of gut microbiota and their metabolic products, though the precise molecular mechanisms require further investigation.

While this study elucidates potential mechanisms underlying CH-mediated suppression of PCa growth through gut microbiota, several limitations should be noted. First, current studies systematically lack dose-response relationship data in evaluating the antitumor effects of cepharanthine hydrochloride (CH). Second, since the research was conducted in murine models, the translational relevance to humans remains to be validated given inherent differences in gut microbiota composition and physiological environments between species. Finally, although multi-omics approaches have unveiled the correlations among the gut microbiota, metabolites, and gene expression, to establish a definitive causal relationship, further *in vivo* and *in vitro* experiments are required to validate the microbiota and their metabolites identified in this study that are strongly associated with CH’s inhibition of prostate cancer.

In conclusion, CH appears to inhibit PCa growth by remodeling gut microbiota in tumor-bearing mice, thereby inducing subsequent changes in intestinal metabolites and transcriptome profiles. This study establishes a novel “microbiota-metabolome-transcriptome” multi-omics regulatory network, providing fresh insights and theoretical foundations for understanding CH’s anti-tumor mechanisms against PCa.

## Data Availability

The original contributions presented in the study are publicly available. The 16s rRNA data can be found here: https://ngdc.cncb.ac.cn/gsa/browse/CRA028639; the RNA-seq data can be found here: https://ngdc.cncb.ac.cn/gsa/browse/CRA028611.
